# Heparin-binding protein improves prediction of severe sepsis in the emergency department

**DOI:** 10.1186/cc12904

**Published:** 2013-11-05

**Authors:** Adam Linder, Ryan Arnold, Marco Zindovic, Igor Zindovic, Anna Lange-Jendeberg, Magnus Paulsson, Patrik Nyberg, Bertil Christensson, Per Åkesson

**Affiliations:** 1 Skåne University Hospital, Lund, Sweden; 2 Cooper University Hospital, Camden, NJ, USA; 3 Örebro University Hospital, Örebro, Sweden; 4 Skåne University Hospital, Malmö, Sweden; 5 Linköping University Hospital, Linköping, Sweden

## Background

The early identification of risk of developing severe sepsis in patients with suspected infection remains a difficult challenge. We hypothesized that an elevated plasma level of heparin-binding protein (HBP), a neutrophil-secreted mediator of vascular leakage, would be a predictor of delayed clinical deterioration and progressive organ dysfunction in emergency department (ED) sepsis patients.

## Materials and methods

A prospective, multicenter study in Sweden and the US was conducted of 763 patients presenting to an ED with a suspected infection and signs of systemic inflammation. Based on recorded clinical and laboratory parameters and final diagnoses, patients were classified into various groups depending on the severity of the infection and inflammatory response. Plasma levels of HBP were measured and compared with levels of other standard sepsis biomarkers including procalcitonin, lactate, WBC, and C-reactive protein.

## Results

The final diagnoses were severe sepsis with organ failure in 338 patients, nonsevere sepsis without organ failure in 340 patients, and no infection in 85 patients. One-hundred and forty-three patients (19%) presented without signs of severe sepsis, but developed delayed circulatory failure and/or organ dysfunction within 72 hours of enrolment. In this patient group, an elevated HBP level could predict the delayed development of severe sepsis with an AUC value of 0.86. Elevated HBP levels (>30 ng/ml) were found in 80% of the patients and elevated procalcitonin levels (>0.5 ng/ml) were detected in 59%, 10.5 hours (median) before developing severe sepsis.

## Conclusions

Detection of elevated plasma-HBP levels may help to provide an early risk-stratification of patients with suspected infections in the ED. An elevated HBP level was independently able to predict delayed clinical deterioration to overt shock or severe sepsis with organ failure.

## Clinical trial number

ClinicalTrials.gov NCT01392508 (the IMPRESSED study).

